# Vacuum-assisted early transanal closure of leaking low colorectal anastomoses: the CLEAN study

**DOI:** 10.1007/s00464-017-5679-6

**Published:** 2017-06-29

**Authors:** W. A. A. Borstlap, G. D. Musters, L. P. S. Stassen, H. L. van Westreenen, D. Hess, S. van Dieren, S. Festen, E. J. van der Zaag, P. J. Tanis, W. A. Bemelman

**Affiliations:** 1 Department of Surgery, Academic Medical Center, University of Amsterdam, PO Box 22660, 1100 DD Amsterdam, The Netherlands; 2 Department of Surgery, Academic Hospital Maastricht, Maastricht, The Netherlands; 3 Department of Surgery, Isala Klinieken, Zwolle, The Netherlands; 4 Department of Surgery, Antonius Zorggroep, Sneek, The Netherlands; 5 Department of Surgery, Onze Lieve Vrouwe Gasthuis, Amsterdam, The Netherlands; 6 Department of Surgery, Gelre Ziekenhuis, Apeldoorn, The Netherlands

**Keywords:** Anastomotic leakage, Rectal cancer, Vacuum therapy, Transanal closure

## Abstract

**Introduction:**

Non-healing of anastomotic leakage can be observed in up to 50% after total mesorectal excision for rectal cancer. This study investigates the efficacy of early transanal closure of anastomotic leakage after pre-treatment with the Endosponge^®^ therapy.

**Methods:**

In this prospective, multicentre, feasibility study, transanal suturing of the anastomotic defect was performed after vacuum-assisted cleaning of the presacral cavity. Primary outcome was the proportion of patients with a healed anastomosis at 6 months after transanal closure. Secondary, healing at last follow-up, continuity, direct medical costs, functionality and quality of life were analysed.

**Results:**

Between July 2013 and July 2015, 30 rectal cancer patients with a leaking low colorectal anastomosis were included, of whom 22 underwent neoadjuvant radiotherapy. Median follow-up was 14 (7–29) months. At 6 months, the anastomosis had healed in 16 (53%) patients. At last follow-up, anastomotic integrity was found in 21 (70%) and continuity was restored in 20 (67%) patients. Non-healing at 12 months was observed in 10/29 (34%) patients overall, and in 3/14 (21%) when therapy started within three weeks following the index operation. Major LARS was reported in 12/15 (80%) patients. The direct medical costs were €8933 (95% CI 7268–10,707) per patient.

**Conclusion:**

Vacuum-assisted early transanal closure of a leaking anastomosis after total mesorectal excision with 73% preoperative radiotherapy showed that acceptable anastomotic healing rates and stoma reversal rates can be achieved. Early diagnosis and start of treatment seems crucial.

Anastomotic leakage is still one of the most dreaded complications following rectal cancer surgery using total mesorectal excision (TME) [1]. Extensive research has focussed on predisposing factors. The common thought is that the leak is being caused by a broad spectrum of both adjustable and non-adjustable factors [2]. Despite optimising surgical techniques (minimal invasive surgery) and perioperative management (ERAS), the leakage rates of colorectal anastomosis remained high (8–20%) over time [2–9]. The TME creates a presacral cavity behind the anastomosis where large amounts of debris and pus can accumulate even in case of a minor anastomotic dehiscence. The anal sphincter functions as a physiologic barrier preventing drainage of the abscess cavity and neorectum via the anus.

Traditionally, if a patient presents with a symptomatic leak, the anastomosis is defunctioned, if not done so primarily. The abscess is most commonly drained either percutaneously or transanally. Half of these leaks might heal with this conventional management [10]. But if these leaks do not heal, a chronic presacral sinus is formed. The sinus might be asymptomatic, but can also be a source of major morbidity even up to life-threatening necrotising fasciitis of the upper leg [11]. Management of the chronic sinus means major surgery taking down the leaking anastomosis followed by either redo anastomosis or intersphincteric proctectomy with omentoplasty and permanent colostomy [12]. The Dutch TME trial showed that after secondary stoma formation for infectious problems, the deviating stoma could not be reversed in 49% with preoperative radiotherapy as an independent predictor (HR 0.34) [13].

Since a chronic sinus requires extensive surgical intervention, it is of great importance to prevent the leak to become a chronic sinus. A minimal invasive treatment strategy with vacuum-assisted drainage (EVAC) combined with early transanal closure of the anastomotic defect (‘vacuum-assisted early transanal closure’) has been very successful in the early management of leaking ileoanal anastomoses for ulcerative colitis (UC) or familial polyposis (FAP) [14]. However, these patients have a neorectum made of small bowel instead of colon, and did not receive neoadjuvant radiotherapy. For this reason we aimed to study the efficacy of vacuum-assisted early transanal closure for rectal cancer patients in terms of anastomotic healing, stoma closure, functionality of the neorectum, quality of life (QoL) and treatment-related costs.

## Methods

This prospective, multicentre, feasibility study, was carried out in a total of five hospitals throughout the Netherlands that performed vacuum-assisted early transanal closure. Between July 2013 and July 2015, eligible patients with an anastomotic leak after TME surgery from the study centre or a referring hospital were included. Patients who underwent TME for rectal cancer with primary anastomosis up to 6 cm from the anal verge and a confirmed leak by either CT-scan or sigmoidoscopy were considered eligible. Prior to start of EVAC, the anastomosis needed to be defunctioned, if not done so at the index operation. During the exchanges of the Endosponge^®^ (B. Braun Medical B.V., Melsungen, Germany), the final decision was made whether closure of the anastomosis was feasible. Closure was considered feasible if the anastomotic edges at the level of the dehiscence were approximating during desufflation with the endoscope. This could be considered as a sign of sufficient flexibility of both sides of the bowel wall to bring the edges together.

Patients that were considered not suitable for closure of the anastomotic defect could proceed with EVAC, thereby gradually tapering the size of the Endosponge^®^. Such patients eventually being treated according to the initial description of EVAC without transanal suturing were excluded from the present study [15]. The Institutional Review Board of the Academic Medical Centre in Amsterdam granted exemption from Ethics approval for this study; however, informed consent was requested for sending out the functionality and quality of life questionnaires.

### Vacuum-assisted early transanal closure

**Fig. 1:**
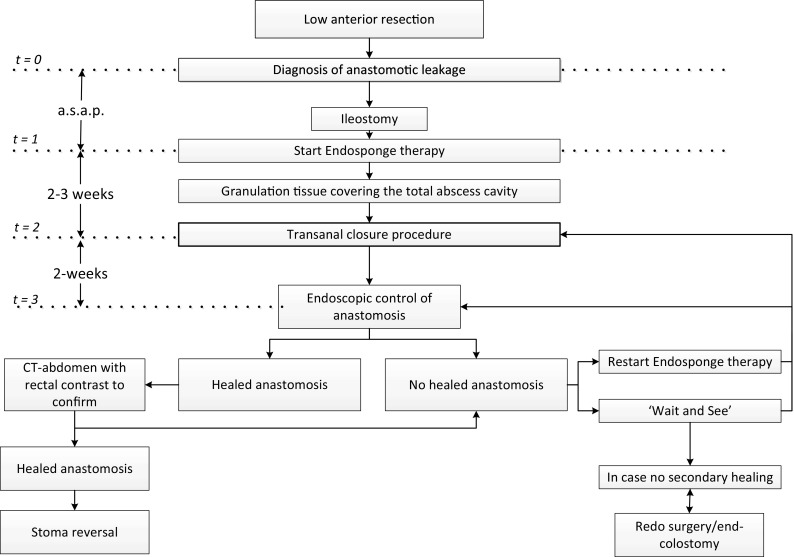
Treatment flow chart of patients with anastomotic leakage after low anterior resection that underwent a vacuum-assisted early closure strategy

Our group described the method of vacuum-assisted early transanal closure for patients with a leaking ileal pouch-anal anastomosis (IPAA) in a previous communication [14, 16]. We refer to those papers for extensive description of the technique [14, 16]. Briefly, the Endosponges^®^ were placed endoscopically under light sedation (dormicum/fentanyl). One or two open-pored polyurethane Endosponges^®^ were placed via a plastic tube under the guidance of the endoscope into the deepest point of the abscess cavity. During subsequent placements, the Endosponges^®^ were not tapered, because the objective of the EVAC therapy was to clean the cavity prior to closure of the anastomotic dehiscence with induction of granulation tissue. This is different from EVAC therapy aiming at closure, where the Endosponges^®^ were gradually tapered in order to achieve a collapse of the cavity [17]. The Endosponges^®^ are connected to a low-vacuum suction bottle (Redyrob^®^ TRANS PLUS suction device, Melsungen, Germany) and changed every 3–4 days to prevent tissue ingrowth. When the abscess cavity was considered clean (i.e. granulation tissue covering the abscess cavity) and bowel edges were expected to come together, the anastomotic defect was closed surgically. Closure of the leak was performed under general anaesthesia with the help of a Lone Star Retractor^®^ (Cooper Surgical, Trumbull, United Stated) or via transanal minimal invasive surgery (TAMIS) using the GelPOINT ^®^ Path Transanal Access Platform (Applied Medical, Rancho Santa Margarita, United States) depending on the distance of the anastomosis from the anal verge. Suturing was done using 2-0 Novosin interrupted sutures (B. Braun Medical B.V., Melsungen, Germany). A drain was placed transanastomotic or perianastomotic in the cavity and removed on the third or fourth postoperative day. Patients were treated with antibiotics up to the tenth postoperative day. The reconstructed anastomosis was evaluated by endoscopic inspection and subsequent contrast imaging studies, two weeks after the transanal closure. If no healed anastomosis was observed at this follow-up visit, either a ‘wait and see’ strategy was chosen or a secondary period of EVAC was commenced, or a second attempt to close the defect transanally, based on the size of the remaining leak. The treatment strategy flow chart is presented in Fig. 1. A member of the steering committee attended at least one transanal closure procedure in the other participating centres. No proctoring courses were organised.

### Definitions

An anastomosis was considered to be healed if there were no signs of contrast extravasation during abdominal CT or contrast enema and there was an intact anastomosis during endoscopy. If patients required redo surgery with excision of the anastomosis with either end colostomy or a new coloanal anastomosis, they were considered as treatment failures. Stoma closure was considered successful if no subsequent pelvic abscess developed during follow-up, if there was no need of recreation of the stoma and if no other complications had occurred related to anastomotic failure. A chronic sinus was defined as a presacral abscess that was still present 12 months onwards after the index operation.

### Outcomes

The primary endpoint was the number of healed anastomoses after 6 months following the first attempt to close the leak surgically. Secondary outcomes were the number of healed anastomoses at the end of follow-up, the number of chronic sinuses, number of patients with successfully restored continuity, quality of life, functionality and direct medical costs. A subanalysis was made between patients that started with EVAC within three weeks (early EVAC group) from the index operation and those where the EVAC started outside this period (late EVAC group), hypothesising that vacuum-assisted early transanal closure is more effective when started early.

### Quality of life and functionality analysis

Quality of life (QoL) and function were assessed at fixed time-points after the index operation. QoL was assessed using the Short Form 36 (SF-36^®^), the GIQLI (Gastrointestinal Quality of Life Index) questionnaire and the EQ-5D-5L at 3, 6, 9 and 12 months postoperative [18–20]. Function was assessed in patients where the continuity was restored, using the Colorectal Functional Outcome (COREFO) scale at 6, 9 and 12 months post construction of the anastomosis and the low anterior resection syndrome (LARS) score at the end of the follow-up period [21, 22].

## Cost analysis

The direct medical costs that were related to vacuum-assisted early transanal closure were calculated from first EVAC to a healed anastomosis. In the case no anastomotic healing occurred, the cost from first EVAC to the decision to definitively refrain from the minimally invasive strategy was calculated (i.e. decision to perform a redo procedure, to construct an end colostomy or decision to definitively not reverse the stoma, also see red dotted line of Fig. 3). Failed patients either had a redo anastomotic pull-through procedure, an intersphincteric resection with permanent colostomy or the intentionally temporary defunctioning ileostomy became permanent. The costs associated with stoma reversal, the redo procedure itself or subsequent complications are not directly associated with vacuum-assisted early closure and are therefore presented in the Appendix 2. Units that were included in the cost analysis were number of EVACs performed, length of stay following transanal closure, readmissions, interventions, total length of hospital days during follow-up, endoscopic examinations, radiological imaging, outpatient clinic visits and emergency room visits. Unit costing was based on the Dutch costing manual for healthcare research. Costs related specifically to the EVAC or stoma-related costs were determined based on earlier cost-analyses from our study group [14, 23]. The unit costs were determined for the year 2015, after price-indexing (based on general consumer price indices; www.cvz.nl, access date 12 October 2016) of unit costs stemming from different calendar years.

### Statistical analysis

According to their distribution, descriptive data are reported as median with range or mean with a standard deviation (SD). As the sample size of this feasibility study was small, no formal comparisons of subgroups were performed. Therefore, no Chi-squared or Fischer’s exact tests were used. In order to analyse the time to healed anastomosis and time to continuity, the Kaplan–Meier method was used. In order to analyse the QoL questionnaires over time, the Wilcoxon-signed rank test was used. All analyses were performed with IBM SPSS statistics, version 23.00 (IBM Corp Armonk, NY, United States).

## Results

**Fig. 2:**
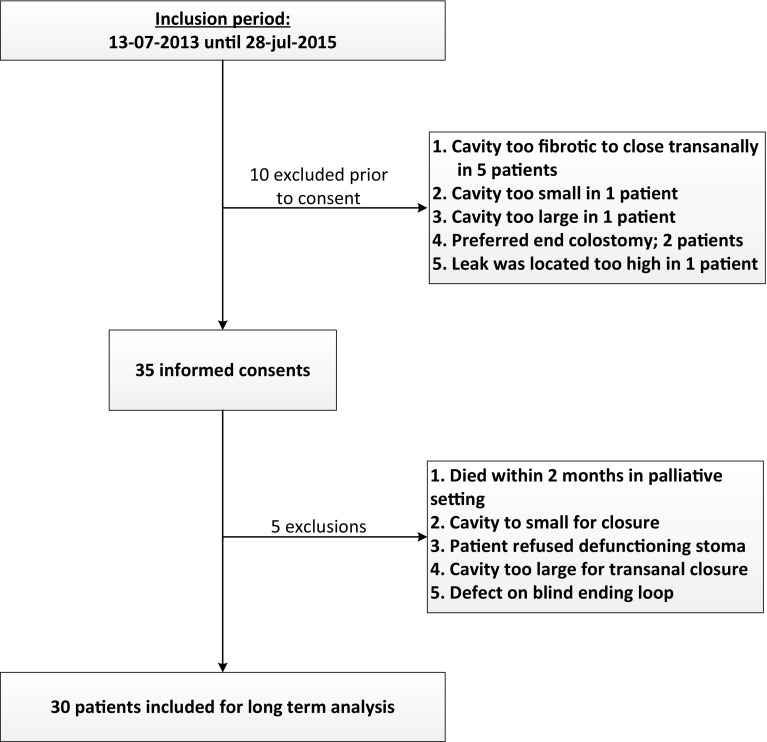
Study flow chart

Between July 2013 and July 2015, a total of 45 patients were counselled for vacuum-assisted early closure. A third of these patients (*n* = 15) were considered not eligible. The patient flow chart and reasons for exclusion are presented in Fig. 2. Finally, a total of 30 patients were included for analysis of the primary endpoint being a healed anastomosis at 6 months from transanal closure.

### Patient characteristics

All patients had prior TME surgery for rectal cancer, of whom 27 patients had a primary leaking anastomosis and three patients had undergone a redo coloanal anastomosis with recurrent leakage. The majority of patients were male, and the median age was 66 (40–79) years. Seven patients were not initially diverted and received a diverting ileostomy after the leak was diagnosed. Any form of neoadjuvant therapy was applied in 22 (73%) patients. Median follow-up was 14 (7–29) months.

The baseline characteristics are presented in Table 1.

**Table 1:** Patient characteristics

	CLEAN study patients (*n* = 30)
Sex (Male)	19 (63%)
Age (median, range)	66 (40–79)
BMI-median (range)	25.2 (20.1–34.2)
Neoadjuvant radiotherapy 5 × 5	19 (63%)
CRT	3 (10%)
Procedure prior to Endosponge^®^ therapy	
LAR	27 (90%)
Anastomotic redo	3 (10%)
Laparoscopic approach	22 (73%)
Earlier abdominal surgery	10 (33%)
Primary deviation	23 (77%)
Stapled anastomosis	26 (87%)
Configuration anastomosis	
Side to end	20 (67%)
End to end	10 (33%)
Median duration of follow-up	14 (7–29)

*CRT* chemoradiotherapy, *LAR* low anterior resection, earlier abdominal surgery that is not related to rectal carcinoma or anastomotic leakage

The leak was located posteriorly in the rectum in 22 patients and a near complete dehiscence of the anastomosis was found in two patients. Median time from the index operation to diagnosis of the leak was 14 (3–75) days. Time from surgery to first Endosponge^®^ placement was median 23 (3–158) days, and EVAC started within three weeks from the index operation in 15 patients. In 9 patients, the defect of the anastomosis needed to be dilated with a 12 mm endoscopic CRE balloon in order to enter the abscess cavity. Transanal closure was performed after a median of 13 (5–51) days and a median of four (2–15) placements. No adverse events related to the EVAC procedures were reported. The details on leakages, EVAC procedures and transanal closures is presented in Table 2.

**Table 2:** Leakage, EVAC and transanal closure characteristics

	CLEAN study patients (*n* = 30)	Start Endosponge^®^ therapy	
		<3 weeks (*N* = 15)	>3 weeks (*N* = 15)
Location of leakage (assessed during transanal closure)			
Ventral	4 (13%)	1 (7%)	3 (20%)
Dorsal	22 (73%)	10 (67%)	12 (80%)
Lateral	2 (7%)	2 (13%)	0
Complete dehiscence	2 (7%)	2 (13%)	0
Median time (days, range) from surgery to diagnose leak	14 (3–75)	9 (3–14)	24 (3–75)
Median time (days, range) from surgery to start Endosponge^®^	23 (3–158)	13 (3–21)	34 (25–158)
Duration Endosponge^®^ therapy prior to early transanal closure in days (median, range)	13 (5–51)	12 (6–51)	13 (5–44)
Number of Endosponge^®^ procedures prior to transanal closure (median, range)	3.5 (2–15)	3 (2–15)	4 (2–13)
Patients requiring Endosponge^®^ post transanal closure	13 (43%)	5 (33%)	8 (53%)
Defect closure confirmed at first imaging endoscopy after transanal surgery (%, *n*)	2 (7%)	1 (7%)	1 (7%)

*Evac* vacuum-assisted drainage

Considering the transanal closures, median operation time was 70 (26–257) min. The defect was closed with the use of a GelPOINT path in 13 (43%) and with a Lone Star Retractor^®^ in 17 (67%) patients. No adverse events related to the transanal closure occurred. The perianastomotic drain was removed after a median of four (2–6) days in the outpatient clinic. Length of hospital stay following the transanal closure was a median of 1.5 (0–21) days.

### Primary endpoint

In 16/30 patients (53%) the anastomosis was healed within 6 months after transanal closure. If EVAC was started within three weeks after the index operation (early EVAC group), anastomotic healing at 6 months was observed in 10/15 (67%) patients, compared to 6/15 (40%) patients with start of EVAC beyond three weeks (late EVAC group). In patients that did not have neoadjuvant therapy, the anastomosis was healed within 6 months in 7 out of 8 patients. Median time for the anastomosis to heal was 127 (14–722) days. At the end of follow-up, the anastomosis had healed in 21/30 (70%) patients. Corresponding rates for the early and late EVAC subgroups were 11/15 (73%) and 10/15 (67%), respectively. The anastomotic leak developed into a chronic sinus in 10/29 (34%) of the patients. In the early EVAC group, 3/14 (21%) patients developed a chronic sinus, compared to 7/15 (47%) in the late EVAC group (Table 3). Interestingly, in three of the patients with a chronic sinus, the anastomosis healed at the end of follow-up.

**Table 3:** Healing rates

	CLEAN study patients (*n* = 30)	Start Endosponge^®^ therapy	
		<3 weeks (*N* = 15)	>3 weeks (*N* = 15)
Healed anastomosis 6 months following transanal closure	16 (53%)	10 (67%)	6 (40%)
Healed anastomosis at end of follow-up	21 (70%)	11 (73%)	10 (67%)
Successfully restored continuity at 6 months	11/30 (37%)	7 (47%)	4 (27%)
Successfully restored continuity end of follow-up (%, *n*)	20/30 (67%)	11 (73%)	9 (60%)
Median time from transanal closure to healed anastomosis (days)	127 (14–722)	92 (19–509)	220 (14–722)
Median time to successful stoma closure from primary surgery (days)	204 (92–624)	193 (92–581)	262 (121–624)
Median time to successful stoma closure from transanal closure (days)	175 (72–556)	175 (72–556)	165 (78–541)
No. of patients with chronic sinus	10/29 (35%)^a^	3/14 (21%)	7/15 (47%)^a^
Number of patients requiring resection of dysfunctional anastomosis (either redo or end colostomies)^b^	6/30 (20%)^b^	2 (13%)	4 (27%)
No. of patients readmissioned for presacral abscess	10/30 (33%)	4 (27%)	6 (40%)
Total hospital days for readmittance in post EVAC + transanal closure course (median, range)^1^	6 (0–47)	6 (0–15)	8 (0–47)

^a^One patient was lost to FU 7 months following the transanal closure and therefore it was unknown whether a chronic sinus had developed

^b^In 3 patients with a chronic sinus, the stoma could be reversed successfully and in 2 patients with a chronic sinus further surgery was declined due to morbidity of the patient in one and widespread metastatic disease in the other, so therefore 5 patients with a chronic sinus were treated conservatively. On the other hand in one patient the anastomosis was resected two weeks after the transanal closure, as the two week sigmoidoscopy showed a complete dehiscence and this was considered to be the best treatment option (however, this patient did not develop a chronic sinus), so therefore the total number is 6. 1 = Includes all readmissions until end of follow-up, thereby including stoma reversals and redo procedures and resection of anastomosis

**Fig. 3:**
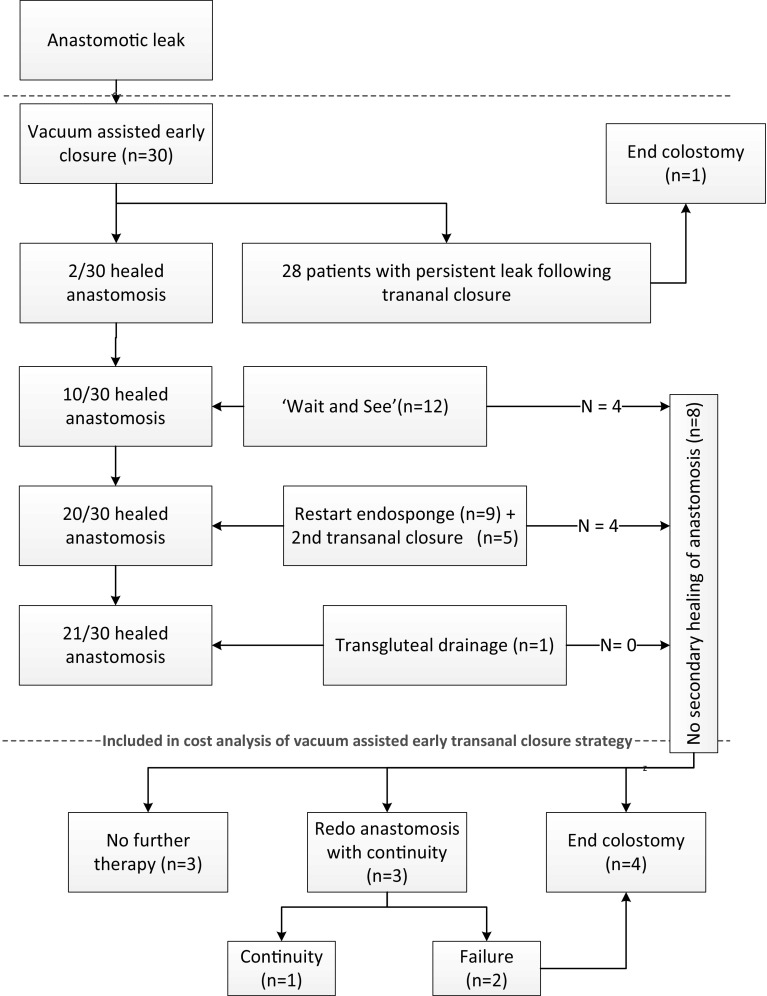
Treatment strategies for patient with a non-healed anastomosis at routine sigmoidoscopy two weeks after transanal closure of the anastomotic defect

Figure 3 presents the strategy applied when no healed anastomosis was observed during sigmoidoscopy at two weeks following the transanal closure. Twenty-eight patients had a persistent leak (93%) at 2 weeks follow-up. In one of these patients, it was decided to take-down the anastomosis because of dehiscence that was larger than 270 degrees. Of the remaining 27 patients, 12 were treated conservatively with monthly sigmoidoscopy follow-up. This strategy was chosen if the remaining dehiscence was too small to restart Endosponge^®^ therapy. In 12 patients, the defect was larger and a second Endosponge^®^ therapy was started. This was followed by a second attempt of transanal closure of the defect in 3 of these 12 patients. In two patients, the persisting defect was considered clean enough, and it was decided to directly attempt a second transanal closure, without pre-treatment with Endosponge^®^. The remaining patient had a presacral abscess without a visible connection with the neorectum, which was successfully treated by percutaneous transgluteal drainage. The corresponding healing rates of these treatment approaches are presented in Fig. 3.

In 19 (67.9%) of the above-mentioned 28 patients, it was possible to save the anastomosis without the need for abdominal surgery. Of the 9 remaining patients, 6 eventually needed a resection of the anastomosis, one refrained from further therapy due to metastatic disease, one patient refused any further therapy and the remaining patient received no further therapy because of poor clinical condition.

### Continuity

The defunctioning ileostomy could be reversed successfully in 20/30 (67%) patients at the end of follow-up. Corresponding rates of the early EVAC group and late EVAC group were 11/15 (73%) and 9/15 (60%), respectively. Reasons for permanent stoma were chronic sinus in seven patients, refusal to have further surgery in two patients and one patient directly received an end colostomy after initial failure of transanal closure. Median interval from transanal closure to stoma reversal was 175 (72–556) days. Corresponding intervals for the early and late EVAC groups were 175 (72–556) and 165 (78–541) days, respectively.

### Quality of life and functionality

The response rates of the EQ-5D-5l, GIQLI and SF-36^®^ at 3, 6, 9 and 12 months were 73, 69, 66 and 86%, respectively. Mean EQ-5D-5L VAS Score at 3 months was 69 (SD 0.22) which increased to 77.9 [SD = 0.25, *p* < 0.01)] at 12 months. A similar improvement in QoL was seen in the SF-36 scale and GIQLI which is presented in Appendix 1. Response rates of the COREFO were 70, 71 and 100% at 6, 9 and 12 months.

With the lower scores corresponding with less continence disturbance, analysis of the COREFO showed that functionality did not increase from 6 to 12 months postoperative. Response rate of the LARS-score was 80%. Of these responders, 81% experienced Major LARS, 13% Minor-LARS and 6% No LARS (Appendix 2).

## Cost analysis

### Direct medical costs related to vacuum-assisted early closure

The total direct medical costs related to the vacuum-assisted early closure strategy from start of EVAC to a healed anastomosis or strategy switch from the minimal invasive treatment strategy were €8933 (95% CI 7268–10,707) per patient. The endoscopic examinations to place the Endosponges^®^ and to monitor the healing of the anastomosis contributed the most to the overall cost-burden with median costs of €1539 (616–6773) per patient. The price of one Endosponge^®^ set was €195, contributing to a median €990 (396–6142) per patient. Compared to earlier communicated results from the ‘wait and see’ strategy in our institution, vacuum-assisted early transanal closure showed a 20% increase in healed anastomoses at the end of follow-up [10]. This resembles a number needed to treat of five. This means that five patients have to be treated with vacuum-assisted early transanal closure in order to save one extra anastomosis compared to the ‘wait and see’ strategy.

## Discussion

Only half of the leaking anastomosis treated with vacuum-assisted early transanal closure had healed at 6 months of follow-up. This was rather disappointing considering the high healing rate of leaking ileoanal anastomoses at 6 months follow-up using the same technique [14].

There are several reasons why this EVAC technique is less successful in colorectal/anal anastomoses than in ileoanal anastomoses (IPAA). First, time interval before initiation of the EVAC was substantially longer (median of 23 days). Most of the patients were referred after a delayed diagnosis of the leakage. Starting too late with EVAC after the primary operation will probably result in fibrotic bowel ends of the leaking anastomosis in such a way that primary closure is more difficult to achieve. This was demonstrated by a difference in success rate between EVAC started before three weeks and after three weeks. In our previous communication on EVAC for leaking IPAA, all patients had their primary surgery in our hospital and therefore a rapid initiation of EVAC after establishment of the diagnosis was possible in nearly all patients. EVAC was commenced after a median of 2 days from diagnosis of leakage in our previous publication compared to a median of 9 days in the CLEAN study cohort.

The second factor causing lower healing rates was that most of the patients with colorectal anastomoses were treated with neoadjuvant radiotherapy, an important cause for disturbed wound healing [24]. Parallel to the impaired wound healing of perineal wounds following abdominoperineal resection, neoadjuvant radiotherapy seems to have a high impact on secondary healing of the anastomosis. The induced cell-death, vascular damage and associated fibrosis all contribute to lower healing rates [24, 25].

Thirdly, it is technically easier to close a leaking IPAA compared to a colorectal/anal anastomosis. The former is easier to access transanally. In this respect, a technical learning curve has to be appreciated in this study with respect to skills, the approach and the type of sutures that are used. However, at the end of follow-up, 70% of the anastomoses healed which is 20% better than the figures found in an earlier published ‘wait and see’ cohort, as well as the Dutch TME trial [10, 13]. The ‘wait and see’ strategy was associated with a high rate of readmissions taking an average of 22 days, whereas in the present cohort the same figure was only 6 days (including readmissions for stoma reversal). This highlights an important benefit of the vacuum-assisted early transanal closure strategy as it facilitates a better local control of the pelvic sepsis. By early sanitising the presacral abscess cavity, the risk on long-term complications requiring readmission seems to be reduced.

A population-based study in the Netherlands demonstrated that the chronic sinus rate following anastomotic leak is around 48% (unpublished data), which is similar to the non-reversal rate of secondary stoma’s in the TME trial. In the early EVAC group, this rate was 21%, and even though our sample size is small, this reduction in chronic sinus rate of 50% is at least promising. On the other hand, the sinus rate of 47% in the late EVAC group is still alarming and questions the applicability of vacuum-assisted early closure after delayed diagnosis of anastomotic leakage.

**Fig. 4:**
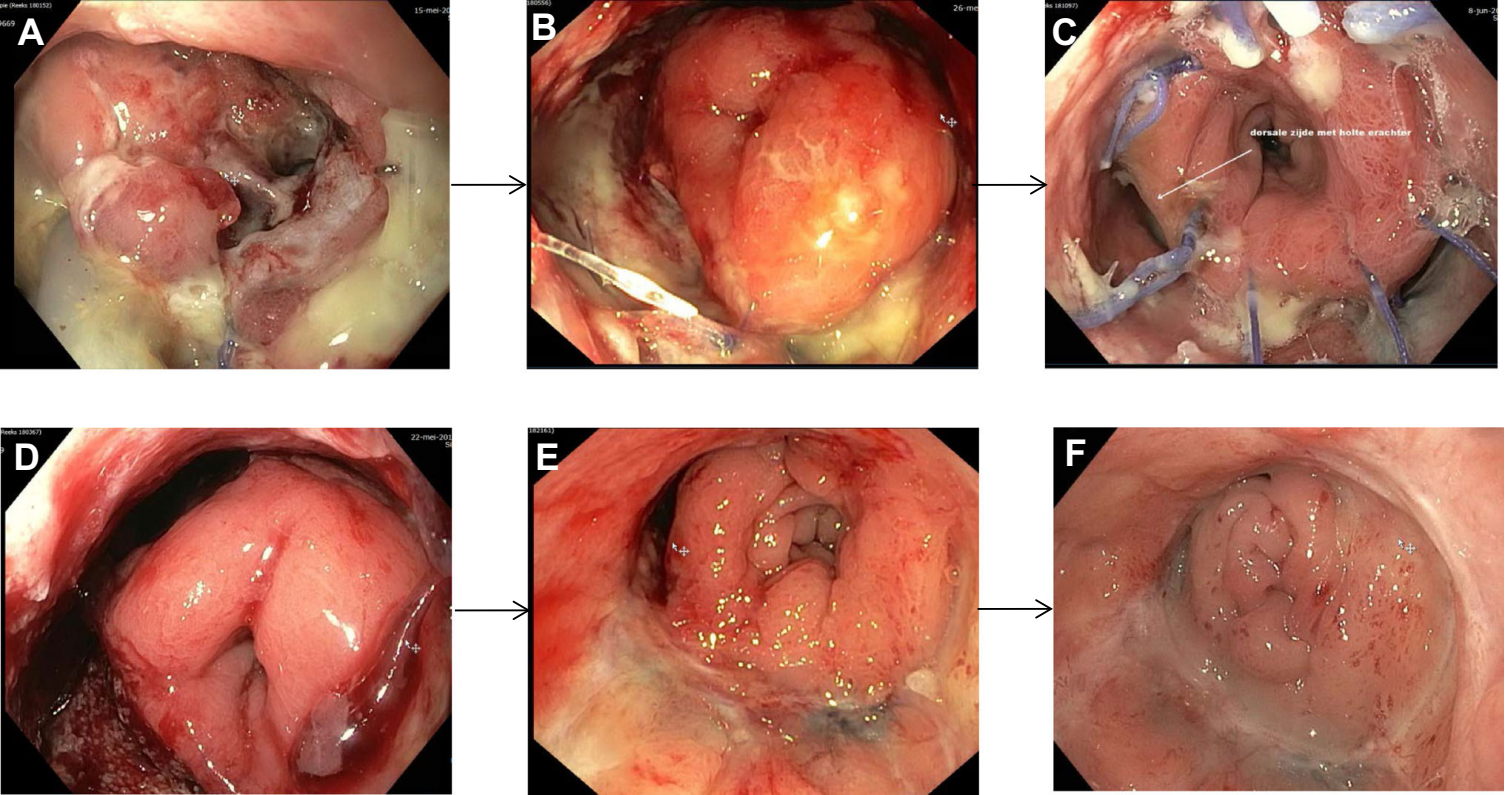
Patient with a large dehiscence of the anastomosis that underwent successful treatment. **A** First sigmoidoscopy showing a 270 degrees dehiscence of the anastomosis with a transanal drain that was placed in the referring hospital. **B** Sigmoidoscopy image after two Endosponge^®^ procedures, showing granulation tissue with pus on the *right side* of the descending colon. **C** Image after the fifth Endosponge^®^ procedure showing a clean cavity with granulation tissue. **D** Two weeks follow-up sigmoidoscopy after transanal closure showing a reduced dehiscence, but with a residual defect. **E** Small residual sinus after a total of 8 Endosponge^®^ exchanges for a residual defect after transanal closure. **F** Sigmoidoscopy two week after the last Endosponge^®^ procedure, showing a healed anastomosis

The primary closure strategy enabled even patients with major dehiscence of the anastomosis (270 or even 360 degrees) to be healed at the end of follow-up due to approximation of the bowel ends which facilitates secondary healing (Fig. 4). A functional anastomosis would have never been possible in such cases without the early surgical intervention, considering the size of the defect. Furthermore, historical data from our institute indicate that the time to heal when applying a ‘wait and see’ strategy doubles when compared to the present cohort [10].

Since the publication of Weidenhagen et al., multiple retrospective studies have published results of the EVAC management of anastomotic leaks [16, 17, 26–28]. Anastomotic healing rates ranged between 56 and 97%, the majority being higher than our reported 70%. In contrast to these studies, our cohort solely consists of patients that had full TME with a low anastomosis rather than partial mesorectal excision and more than 70% had preoperative radiotherapy.

An important limitation of the present study is the risk of selection bias. The period needed to include 30 patients was rather long, despite the CLEAN study had been presented at several national meetings and surgeons were invited to refer their leakages through different types of communications. Maybe not all potential study candidates were counselled and included in the study. Most likely, the surgeons referred their worst cases for inclusion expecting no healing by applying a wait and see policy. Another limitation is the lack of a comparative group. This cohort study was designed as proof of concept of a technique that has been shown to be successful for another indication, with the ability of historical comparison with published results from the ‘wait and see’ strategy.

The minimally invasiveness of EVAC, the low-associated costs and the low risks associated with the transanal closure procedure itself, advocate vacuum-assisted early transanal closure as a first step in a proactive and step-up approach of anastomotic leak management. In case no anastomotic healing is reached with vacuum-assisted transanal closure, eventually a redo anastomotic pull-through procedure can be considered [29]. Continuity after redo surgery can be reached in approximately 70–80% of the patients [29, 30]. Another slightly less complex management of the chronic sinus is performing an intersphincteric completion proctectomy with omentoplasty and a permanent end colostomy [12].

This step-up approach of the anastomotic leak is an extensive treatment strategy that is demanding for patients and physicians. Therefore it is of pivotal importance to include the patient in the decision-making process in each of these steps. Patients should be well informed on the increased risk on intra- and postoperative complications of the surgical procedures itself, but especially on the risk of impaired postoperative functionality of the neorectum. Anastomotic leakage, redo surgery and neoadjuvant therapy are responsible for even more LARS than the procedure is causing itself. This was shown in our cohort as a major LARS-score was observed in those who had preserved bowel continuity.

In conclusion, this first prospective study on vacuum-assisted early closure of a leaking anastomosis following TME surgery and 73% neoadjuvant radiotherapy showed that acceptable anastomotic healing and stoma reversal rates can be achieved with this treatment strategy. However, earlier diagnosis of the anastomotic leak and initiation of EVAC as soon as possible might still improve the success rate of the technique.

## Appendix 1: Quality of life assessment

**Table:**

EQ-5D-5L	3 m (*n* = 19)	6 m (*n* = 20)	9 m (*n* = 18)	12 m (*n* = 24)
EQ-5D index score (mean sd)	0.69 (0.22)	0.79 (0.14)	0.80 (0.17)	0.80 (0.25)
EQ-5D VAS score	67.22 (20.50)	70.26 (14.07)	76.39 (17.05)	77.91 (16.64)

SF-36	3 m (*n* = 19)	12 m (*n* = 25	*p* value*
Physical functioning (mean, SD)	60 (20)	75 (26)	0.031
Role Physical (mean, SD)	25 (35)	66 (46)	0.003
Bodily Pain (mean, SD)	69 (24)	74 (29)	0.312
Social functioning (mean, SD)	53 (34)	71(30)	0.044
Mental health (mean, SD)	71 (25)	78 (19)	0.171
Role emotional (mean, SD)	53 (47)	68 (47)	0.335
Vitality (mean, SD)	55 (22)	65 (26)	0.162
General health perception (mean, SD)	59 (19)	63 (25)	0.569

## Appendix 2: Treatment-dependent direct medical costs per patient of both treatment methods in Euros

**Table:**

Cost analysis	Price per unit (€)	Units	Costs
Costs made from diagnosis of leakage to healed anastomosis or strategy switch (*n* = 30)			
Hospital admittance (day) following transanal closure^1,a^	476	3 (1–19)	1190 (476–9044)
Total hospital days for readmittance in post EVAC course^b^	476	0 (0–21)	0 (0–9996)
Endosponge set (per change)^2^	195	5 (2–31)	990 (396–6142)
Total amount of transanal closures^3^	1413	1 (1–3)	1436 (1436–4307)
Sigmoidoscopy^4^	202	7.5 (3–33)	1539 (616–6773)
CT-abdomen^5^	212	2 (0–4)	431 (0–862)
Colonogram^6^	150	0 (0–6)	(0–902)
Outpatients clinic visits	113	0.5 (0–14)	57 (0–1584)
ER room visits^7^	259	0 (0–2)	0 (0–518)
Reinterventions^#^	378–1915	1 (1–2)	1096 (149–1916)
Total costs^d^			Mean (CI) : 8933-(7268–10707)
CLEAN costs including stoma reversal, redo procedure or proctectomy with end colostomy *(n* = 30)			
Total hospital days for readmittance in post EVAC course	476	6 (0–47)	2856 (0–22,372)
Endosponge set (per change)^2^	195	5 (2–31)	991 (396–6142)
Sigmoidoscopy^4^	202	8 (3–34)	1642 (616–6978)
CT-abdomen^5^	212	2 (0–7)	431 (0–1508)
Colonogram^6^	150	0 (0–6)	0 (0–902)
Outpatients clinic visits	113	1 (0–16)	113 (0–1810)
ER room visits^7^	259	0 (0–2)	0 (0–518)
Reinterventions^#^	378–1915	2 (0–7)	1345 (149–3622)
Redo procedure	5440	0 (0–2)	0 (2–10,880)
Stoma reversal^@^	2504	1 (0–2)	2504 (0–6950)
Total costs^d^			Mean (CI): 17,018 (13,822–20,832)

Data are presented as median (range)

^a^Hospital stay from day of transanal closure to discharge

^b^Hospital stay for readmissions in period from defect closure to healing of anastomosis of strategy switch

^c^Hospital stay for readmissions in period from defect closure including ileostomy reversal, redo procedures and formation of end colostomy

^d^Means with 95% CI are displayed, Unit costing was based on the Dutch costing manual for health care research [15]

^1,2,3,4,5,6,7,#^Other reinterventions than stoma reversal or redo procedure

^@^In the case stoma reversal was complicated due to a dysfunctional anastomosis, the costs of the secondary stoma construction were calculated within the stoma reversal costs

## References

[1] Heald RJ, Ryall RD (1986). Recurrence and survival after total mesorectal excision for rectal cancer. Lancet.

[2] McDermott FD, Heeney A, Kelly ME, Steele RJ, Carlson GL, Winter DC (2015). Systematic review of preoperative, intraoperative and postoperative risk factors for colorectal anastomotic leaks. Br J Surg.

[3] Vermeer TA, Orsini RG, Daams F, Nieuwenhuijzen GA, Rutten HJ (2014). Anastomotic leakage and presacral abscess formation after locally advanced rectal cancer surgery: incidence, risk factors and treatment. Eur J Surg Oncol.

[4] Trencheva K, Morrissey KP, Wells M, Mancuso CA, Lee SW, Sonoda T (2013). Identifying important predictors for anastomotic leak after colon and rectal resection: prospective study on 616 patients. Ann Surg.

[5] Hallbook O, Sjodahl R (1996). Anastomotic leakage and functional outcome after anterior resection of the rectum. Br J Surg.

[6] Maggiori L, Bretagnol F, Lefevre JH, Ferron M, Vicaut E, Panis Y (2011). Conservative management is associated with a decreased risk of definitive stoma after anastomotic leakage complicating sphincter-saving resection for rectal cancer. Colorectal Dis.

[7] Nesbakken A, Nygaard K, Lunde OC, Blucher J, Gjertsen O, Dullerud R (2005). Anastomotic leak following mesorectal excision for rectal cancer: true incidence and diagnostic challenges. Colorectal Dis.

[8] Peeters KC, Tollenaar RA, Marijnen CA, Klein Kranenbarg E, Steup WH, Wiggers T (2005). Risk factors for anastomotic failure after total mesorectal excision of rectal cancer. Br J Surg.

[9] Vlug MS, Wind J, Hollmann MW, Ubbink DT, Cense HA, Engel AF (2011). Laparoscopy in combination with fast track multimodal management is the best perioperative strategy in patients undergoing colonic surgery: a randomized clinical trial (LAFA-study). Ann Surg.

[10] van Koperen PJ, van der Zaag ES, Omloo JM, Slors JF, Bemelman WA (2011). The persisting presacral sinus after anastomotic leakage following anterior resection or restorative proctocolectomy. Colorectal Dis.

[11] Sloothaak DA, Buskens CJ, Bemelman WA, Tanis PJ (2013). Treatment of chronic presacral sinus after low anterior resection. Colorectal Dis.

[12] Musters GD, Borstlap WA, Bemelman WA, Buskens CJ, Tanis PJ (2016). Intersphincteric completion proctectomy with omentoplasty for chronic presacral sinus after low anterior resection for rectal cancer. Colorectal Dis.

[13] den Dulk M, Smit M, Peeters KC, Kranenbarg EM, Rutten HJ, Wiggers T (2007). A multivariate analysis of limiting factors for stoma reversal in patients with rectal cancer entered into the total mesorectal excision (TME) trial: a retrospective study. Lancet Oncol.

[14] Gardenbroek TJ, Musters GD, Buskens CJ, Ponsioen CY, D’Haens GR, Dijkgraaf MG (2015). Early reconstruction of the leaking ileal pouch-anal anastomosis: a novel solution to an old problem. Colorectal Dis.

[15] Weidenhagen R, Gruetzner KU, Wiecken T, Spelsberg F, Jauch KW (2008). Endoluminal vacuum therapy for the treatment of anastomotic leakage after anterior rectal resection. Rozhl Chir.

[16] van Koperen PJ, van Berge Henegouwen MI, Rosman C, Bakker CM, Heres P, Slors JF (2009). The Dutch multicenter experience of the endo-sponge treatment for anastomotic leakage after colorectal surgery. Surg Endosc.

[17] Weidenhagen R, Gruetzner KU, Wiecken T, Spelsberg F, Jauch KW (2008). Endoscopic vacuum-assisted closure of anastomotic leakage following anterior resection of the rectum: a new method. Surg Endosc.

[18] Walters SJ, Brazier JE (2005). Comparison of the minimally important difference for two health state utility measures: EQ-5D and SF-6D. Qual Life Res.

[19] Aaronson NK, Muller M, Cohen PD, Essink-Bot ML, Fekkes M, Sanderman R (1998). Translation, validation, and norming of the Dutch language version of the SF-36 Health Survey in community and chronic disease populations. J Clin Epidemiol.

[20] Eypasch E, Williams JI, Wood-Dauphinee S, Ure BM, Schmulling C, Neugebauer E (1995). Gastrointestinal quality of life index: development, validation and application of a new instrument. Br J Surg.

[21] Bakx R, Sprangers MA, Oort FJ, van Tets WF, Bemelman WA, Slors JF (2005). Development and validation of a colorectal functional outcome questionnaire. Int J Colorectal Dis.

[22] Juul T, Ahlberg M, Biondo S, Emmertsen KJ, Espin E, Jimenez LM (2014). International validation of the low anterior resection syndrome score. Ann Surg.

[23] CVZ. Handleiding voor kostenonderzoek (Manual for Cost Research). College voor zorgverzekeringen, Rotterdam

[24] Bullard KM, Trudel JL, Baxter NN, Rothenberger DA (2005). Primary perineal wound closure after preoperative radiotherapy and abdominoperineal resection has a high incidence of wound failure. Dis Colon Rectum.

[25] Musters GD, Sloothaak DA, Roodbeen S, van Geloven AA, Bemelman WA, Tanis PJ (2014). Perineal wound healing after abdominoperineal resection for rectal cancer: a two-centre experience in the era of intensified oncological treatment. Int J Colorectal Dis.

[26] Arezzo A, Verra M, Passera R, Bullano A, Rapetti L, Morino M (2015). Long-term efficacy of endoscopic vacuum therapy for the treatment of colorectal anastomotic leaks. Dig Liver Dis.

[27] Kuehn F, Janisch F, Schwandner F, Alsfasser G, Schiffmann L, Gock M (2016). Endoscopic vacuum therapy in colorectal surgery. J Gastrointest Surg.

[28] Keskin M, Bayram O, Bulut T, Balik E (2015). Effectiveness of endoluminal vacuum-assisted closure therapy (Endosponge) for the treatment of pelvic anastomotic leakage after colorectal surgery. Surg Laparosc Endosc Percutaneous Tech.

[29] Borstlap WA, Harran N, Tanis PJ, Bemelman WA (2016). Feasibility of the TAMIS technique for redo pelvic surgery. Surg Endosc.

[30] Pitel S, Lefevre JH, Tiret E, Chafai N, Parc Y (2012). Redo coloanal anastomosis: a retrospective study of 66 patients. Ann Surg..

